# Femoral Stem Fracture and Successful Reimplantation Using Femoral Window Technique in Canine Cemented Total Hip Replacement: Case Report

**DOI:** 10.3389/fvets.2021.716297

**Published:** 2021-10-06

**Authors:** Beata Degórska, Jacek Sterna, Magdalena Kalwas-Śliwińska

**Affiliations:** Department of Small Animal Diseases and Clinic, Institute of Veterinary Medicine, Warsaw University of Life Sciences, Warsaw, Poland

**Keywords:** THR complication, dog, femoral component fracture, hip dysplasia, hip prosthesis

## Abstract

A fractured stem is a very rare, late complication in total hip replacement procedure in dogs. Here, we present one case after cemented total hip replacement with successful reimplantation, including clinical signs and radiographic findings.

## Introduction

Total hip replacement (THR) is a widely used surgical treatment of hip dysplasia in dogs and humans. The procedure is applied in case of pathological changes in the hip joint of small and large dog breeds, in both young and adult animals. The main indications include hip dysplasia, osteoarthrosis of the hip joint secondary to hip dysplasia, luxation, osteoarthritis secondary to trauma, Legg–Calves–Perthes disease, slipped capital epiphysis, and femoral head and neck ostectomy revision (1–4). There are two main systems of hip prosthesis available in the veterinary field—cemented and cementless; sometimes a hybrid, i.e., a combination of them, is used (4, 5). The cemented one has been used since 1976 (3, 6), while the cementless one has been used since the 1990's (7, 8).

The main reason for developing the cementless procedures and setting new direction for research on the shape of the stem and the way of fixing it was the complications arising from the use of cement such as aseptic loosening, infections, and extraosseus cement granuloma formation (9).

Several improvements in cemented and cementless fixations, designed to decrease postoperative complications, have been proposed, but some of the complications such as aseptic loosening, infections, and luxation are still present (10, 11).

The reported rate of complications related to the use of THR techniques varies from 3% up to even 56% depending on the publication and chosen criteria (4, 12–19). The most common complications for both the cemented and cementless procedure are luxation, acetabular cup displacement, infection, stem or cup aseptic loosening, femoral fractures, acetabular fractures, subsidence (in some systems), and sciatic neurapraxia. On the other hand, the complications described specifically for the case of the cemented procedure include aseptic loosening, cement granuloma, and pulmonary embolism (3, 9–12, 20–24).

With time it appears that neither of the procedures is problem-free, and neither of them can be seen as superior to the other.

A very uncommon complication is damage to the stem. It has been documented in human medicine and described in case of Zurich cementless total hip system prosthesis (25, 26). For the cemented procedure only two reports exist in the veterinary literature with subsequent implants removal (11, 12).

In this report we describe a clinical case of femoral stem fracture that took place 2 years after the cemented THR with successful reimplantation.

## Narrative

A 2-year-old male Golden Retriever weighing 30 kg underwent cemented THR of the left hind limb due to hip dysplasia and problems with normal activity (exercise intolerance). The arthroplasty involved implantation of the cemented modular Porte S.A. prosthesis: a high-molecular-weight polyethylene acetabular cup (16/25.4) and a modular cobalt-chrome femoral component with independent head and stem (head 16/0; femoral stem 7.5). Both components, i.e., cup and stem, were secured with polymethylmethacrylate (Surgical Simplex® P, Howmedica International, Ireland). Bone cement was slowly mixed in a bowl and inserted into the femur canal with the aid of negative pressure. To achieve the negative pressure, a hole in a midshaft of a femur was drilled with a 4.5 drill-bit, and a suction tip was placed in it. Subsequently, bone cement was inserted into the medullary canal from the proximal femoral approach, pushed down, and sucked down by the application of negative pressure in the midshaft of the femur until the medullary canal was filled.

**Figure 1:**
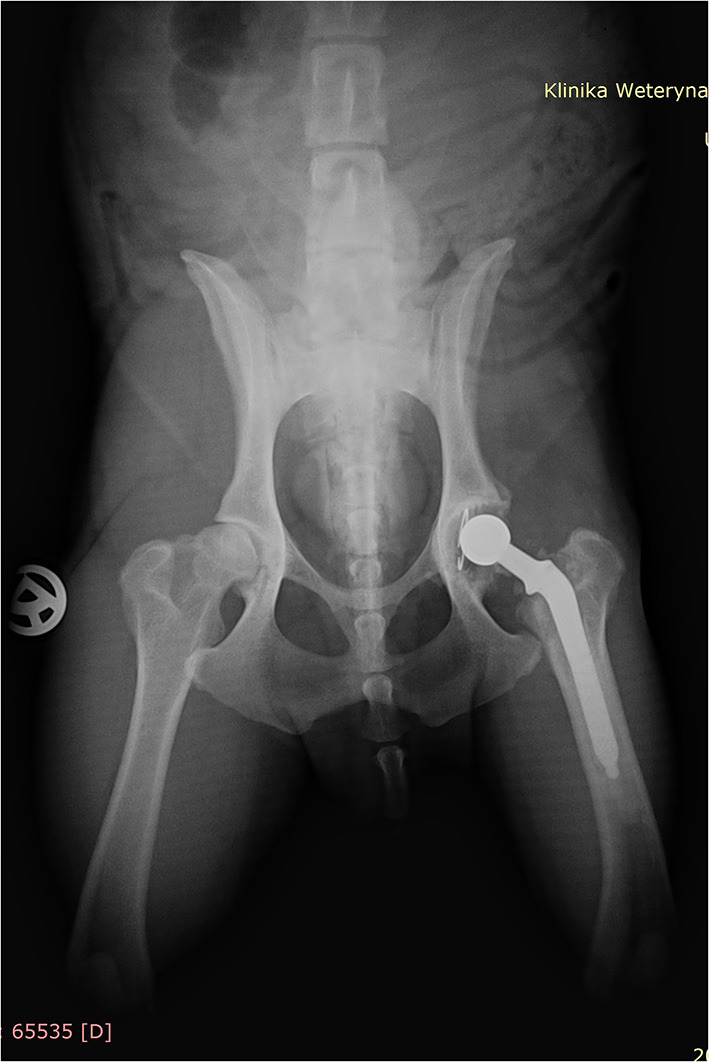
Ventrodorsal radiograph of the dog 2 years after cemented total hip replacement (left hip); radiopaque cement mantle surrounding the femoral and acetabular component is visible.

A post-operative radiographic assessment revealed proper implant's size and positioning. The second radiographic control was performed 3 months after surgery with subsequent yearly clinical examinations, and follow-up was uneventful until 2 years after surgery when the dog started limping on the operated leg. It was the first-degree lameness that was observed occasionally. The owner did not report any trauma. An orthopedic examination of the patient was carried out to evaluate the problem. No changes were found in the ventrodorsal x-ray examination of the hip's joint compared to the examination made the year before (Figure 1). The dog was treated with 2–3 weeks of activity restriction and nonsteroidal pain relief without any clear improvement. During that period the degree of lameness remained unchanged, evident from time to time only in trot. The owner decided to wait and observe the dog.

**Figure 2:**
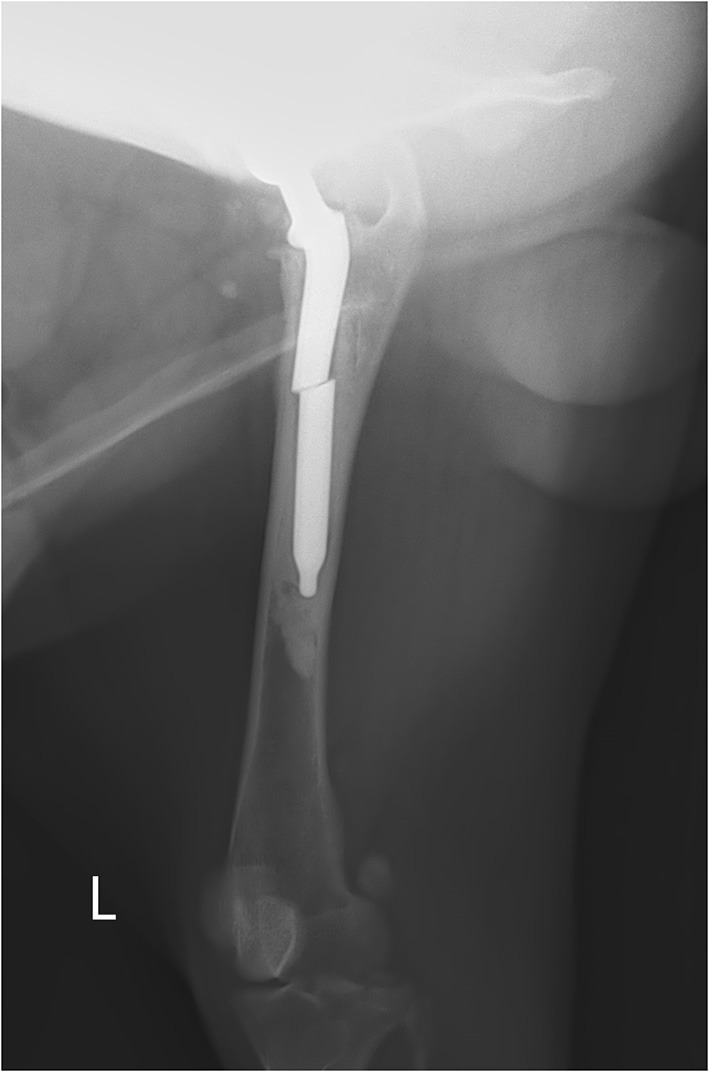
Lateral radiograph with stem fracture in the midshaft—visible only in the lateral view. There is a dislocation in the middle part of the stem with a gap between the cuff cement and the stem, with mild periosteal reaction.

Two months later, the x-ray re-examination in lateral and ventrodorsal projections of the hips demonstrated visible stem fracture in the midshaft. It was well visible only on the lateral view as a step in the middle part of the stem (Figure 2). The cortical bone re-modeling was slightly visible on the level of the stem fracture in the cranial part of the femur with the formation of the new periosteal bone. There was also a noticeable gap between the cuff cement and the stem and a thin gap between the cement mantle and the femoral bone below the major trochanter.

After an additional 2 months, the owner eventually decided to re-operate the dog. In comparison to the previous examination, the x-ray in lateral recumbency showed a bigger dislocation in stem fracture with a huge re-modeling of cortical bone in the area in which the proximal part of the fractured stem irritated the medullary canal and cortical bone. The tip of the distal part of the fractured stem was displaced caudally and remained in contact with the caudal femoral cortex. There was no radiological evidence of a cracked cement mantle. The dog underwent re-implantation of the femoral component using the femoral window technique under general anesthesia. The patient was positioned in lateral recumbency, and a large craniolateral approach was made to allow proper access to the left femur. Deep gluteal muscle tenotomy was made, and capsulotomy with T-shape incision was done. A sample from the joint capsule was collected for microbiological examination. Subsequently, the hip was luxated, and the femur was externally rotated. A rectangular shape of the bone window was made on the lateral side of the femur using a sharp 10-mm-width osteotome. The distal border was extended to the distal cement mantle which was previously determined based on an x-ray. The edges of the bone fragment cut were slightly slanted to prevent further collapse during the planned reposition. As in the original technique, the excised window was less than one-third of the femoral circumference. All the cement inside the femoral canal was fragmented using a mallet, an osteotome, and rongeurs, extracted and removed together with the fractured stem. The medullary canal was debrided and the material for microbiological culture from the canal collected. The osteotomy site was repaired using two cerclage wires, which were hand twisted. The fractured stem was replaced with a new one, the same size (femoral stem 7.5) and secured with polymethylmethacrylate (Surgical Simplex® P, Howmedica International, Ireland). The acetabular component was stable and therefore was left intact at its place. The surgical wound was closed in a routine manner, paying attention to the proper suturing cut tendon of the deep gluteal muscle. Lincomycin with spectinomycin (Linco-spectin®100, Zoetis) was administered intramuscularly after surgery and continued for 7 days.

**Figure 3:**
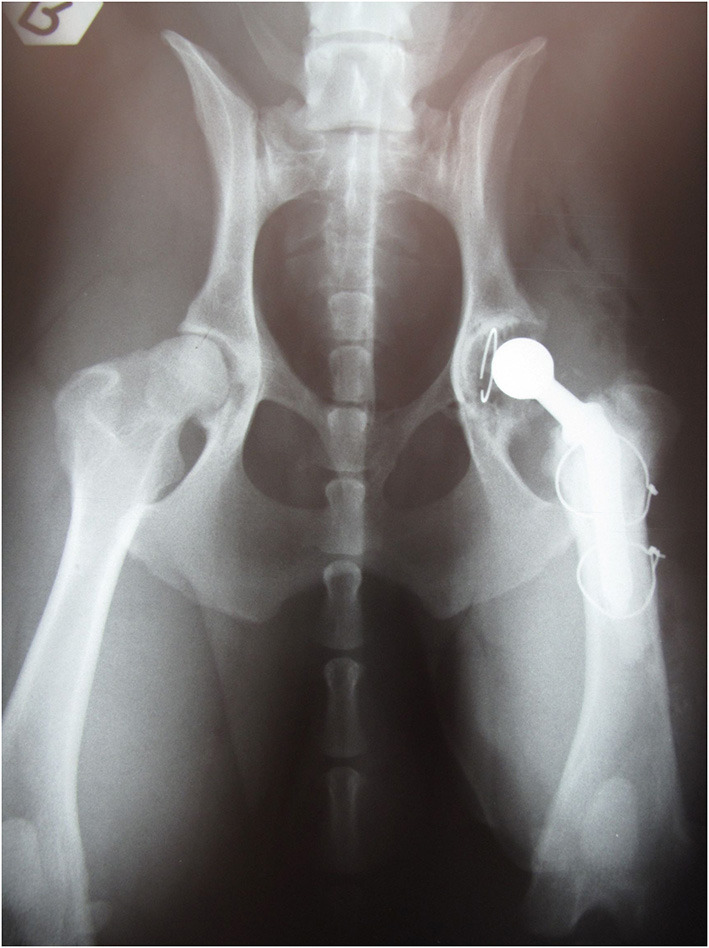
Ventrodorsal radiographs after reimplantation. Proper positioning and orientation of the stem and proper mantle cement are visible. Two cerclage wires were used to stabilize the bone window fragment.

**Figure 4:**
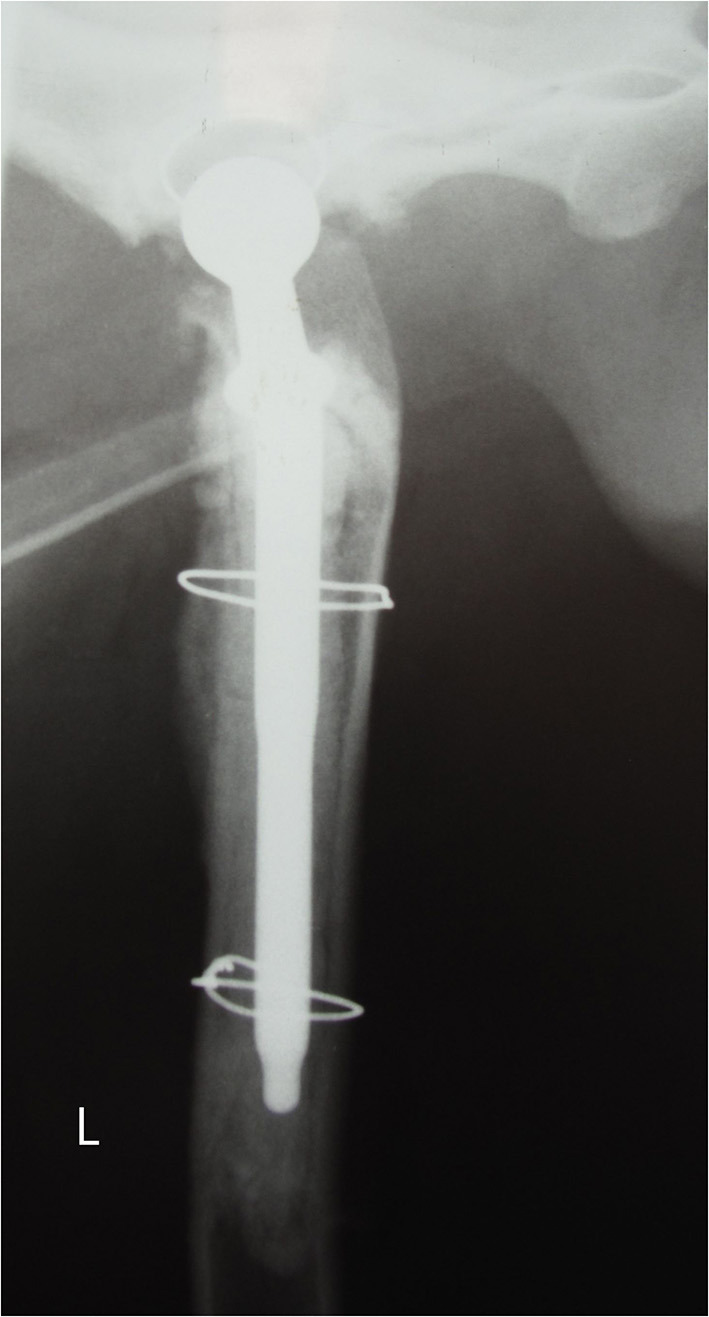
Lateral radiograph 6 months after reimplantation. Proper positioning of the stem and periosteal reaction is visible.

Post-operative lateral and ventrodorsal radiographs of the pelvis were assessed for implant positioning and orientation and to ensure the complete filling of the medullary canal by the cement. The positioning of the implant was correct, and the bone fragment cerclage wires appeared stable (Figure 3). Radiography was repeated at 8 weeks and at 6 months (Figure 4) post-operatively and recommended yearly.

Recovery from surgery was uncomplicated. The surgery wound healed without any problems. The results of the culture—from both samples taken from the acetabular region and femoral canal—were negative.

There was no need for any rehabilitation program because the dog started to walk normally without limping a few days after revision surgery. Restricted activity, i.e., a regular leash walk, was recommended for 8 weeks. The dog was examined annually, and the orthopedic examination was performed by a doctor at the patient's residence. Clinical examinations were done without x-ray examinations because the owner refused yearly x-ray checking. Over 9 years follow-up the dog was normally active without lameness and was put down at the age of 13 due to reasons unrelated to surgery.

## Discussion

THR is a well-established method for the treatment of hip-joints diseases in humans and animals. However, it still faces different types of complications. The rates of these complications depend on the implant system used, surgical technique, surgical experience, and the length of follow-up (10, 11, 16, 24, 25, 27–29).

In human medicine, femoral stem fracture has been reported as a result of suboptimal implant's design, metallurgical composition, quality of cement mantle, and surgical technique. The estimated occurrence is 0.23–0.27%. In human medicine, factors predisposing to stem fracture include also increased stress due to undersized implant, body weight and activity, insufficient cement mantle, technical errors during surgery, and implant's type and design (25).

In veterinary literature, a stem fracture is well known and described for the cementless Zurich THR (26, 29, 30), but reports of femoral component fracture in cemented hip prosthesis are extremely rare (4, 11, 12).

There are four models describing the damage to the stem including axial stem movement, mid-stem or calcar pivoting, and cantilever bending (12). Progressive loosening of proximal cement mantle and subsequent cantilever bending leading to stem fracture was found in 62% of 58 human cases (25). The reason for stem damage includes also undersized implant, which became aseptically loose at the interface between the cement and a bone with subsequent formation of cement granuloma (11, 12). Surgical treatment of these cases of cemented stem fracture consisted of removal of the proximal unstable part of stem prosthesis without reimplantation of a new one.

The situation in the reported case was different; the dog underwent the first surgery being adult, and a proper stem length and diameter, suitable for the medullary canal diameter and the size and weight of the dog, was used. The implant fracture was detected before a significant change in the femur occurred. The patient was in an exceptionally good condition with good muscle mass of the operated leg. During revision surgery, the whole bone cement was removed, and the medullary canal was debrided. A bone structure visible on the x-ray and during revision surgery encouraged reimplantation. The same cemented stem size as the removed one was used. The dog has used the leg without any problems for an impressive time of 9 years after reimplantation. It means that the undersized implant was probably not the main cause of fracture.

Loosening of proximal cement mantle is possible when bone osteolysis appears. This is known as aseptic loosening which is a long-term complication in the cemented THR. This biological process starts months or years after arthroplasty, and it is associated with multiple factors such as implant's design, the orientation of prosthesis components, cementing technique, stress shielding, revascularization, and wear debris (27, 29). It is called “wear debris-mediated osteolysis” due to important components of pro-inflammatory cytokines; induction of fibroblast, phagocyte, and lymphocyte apoptosis; and osteoclast differentiation resulting in reabsorption of a bone matrix (4, 12, 23, 27, 31). The main problem of the slow, progressive process of loosening is that signs and symptoms of the damage are visible when the remodeling of bone is advanced or in the late stage of failure, resulting in a greater cortical area in the proximal part as well as substantial femoral and/or acetabular bone resorption (27).

This phenomenon appears on the border between the implant and bone, between mantle cement and bone, or as a combination of them (4, 23, 31). In one study, aseptic instability was found in 63.2% of 38 postmortem retrieved femoral bones (32).

Another mechanism of loosening is also known and is associated with elastic modulus mismatch between the implant, polymethylmetacrylate (PMMA), and bone (or combination of them), leading to slow degradation in osteointegration (7).

Surgical procedure and technique with stem insertion in the medullary canal lead to a femoral adaptive response to the implant resulting in a decreased cortical bone mass proximally and an increased cortical bone mass distally to the stem. In an unstable prosthesis, mechanical factors such as cracking cement mantle play important roles in implant loosening because of the circumferential stress within the cement and resorption of the calcar (27). The implant position in the medullary canal, implant type, and quality of the cement mantle is also important.

A femoral window technique described by Dyce and Olmstead in 2002 (33) allows getting access to the medullary canal to remove the cement mantle and the stem in revision surgery due to infection. In the reported case, we decided to remove the fractured stem and to replace it with a new one using PMMA. The stem size was the same as the one used during the first surgery. A fibrous membrane between the bone and cement mantle was not found. The presence of this membrane, also known as a synovial-like membrane or fibrous pseudocapsule, usually indicates an aseptic loosening problem (27, 34). This membrane contains and releases mediators of bone lysis, activated macrophages, tumor necrosis factor-α, and oxygen-derived free radicals. It is believed that the presence of this fluid contributes to the extension of the interface and increases the pressure between the bone and the implant (34).

Failure of the femoral stem in the cemented procedure is likely a consequence of fatigue in the stem in a situation in which the distal part of the implant is rigidly fixed into the cement mantle while the proximal one is not rigidly stable. This was probably the cause of the problem in the case described in this report. Loosened border between the cement mantle and the bone in the proximal part of a femur due to aseptic loosening, lack of cortical support, and the stress associated with stem overloads may lead to fatigue of the stem material. Slow loosening of the proximal cement mantle and subsequent cantilever bending leads to stem fracture.

Mechanical and biological factors influence each other. Improper positioning of the femur stem or improper femoral canal filling with PMMA leads not only to micromotions but to the production of wear debris as well.

## Conclusion

When stem failure occurs, there are two options: replacement of the femoral component or removal of the prosthesis. Several factors should be taken into consideration before making the decision of re-implantation. They include the absence of infection, condition of the bone, duration of the process, surgical techniques and surgeon experience, type of prosthesis, general condition of the animal, and the owner's expectations. Revision of unstable prostheses is necessary, and in many cases it is a salvage procedure (11, 12, 27, 33). Revision of fractured cemented stem with implantation of a new one has not been documented so far. The reported case shows that this method with the same size of the stem can be successfully used in other adult dogs qualified for reimplantation of cemented stem as it does not lead to any significant changes in the femur bone tissue. This paper has some limitations. The main one is the lack of long-term follow-up radiographs discussed, but it should not be an obstacle in presenting this clinical case as it is the first report of such complication in cemented techniques with successful reimplantation instead of explantation of the stem.

## Data Availability Statement

The original contributions presented in the study are included in the article/supplementary material, further inquiries can be directed to the corresponding authors.

## Ethics Statement

This case report describes the clinical case and the only needed approval for medical treatment was the owner agreement. Written informed consent was obtained from the owners for the participation of their animals in this study.

## Author Contributions

BD and JS followed the clinical case. BD, JS, MK-S wrote the manuscript. All authors read and approved the final manuscript.

## Conflict of Interest

The authors declare that the research was conducted in the absence of any commercial or financial relationships that could be construed as a potential conflict of interest.

## Publisher's Note

All claims expressed in this article are solely those of the authors and do not necessarily represent those of their affiliated organizations, or those of the publisher, the editors and the reviewers. Any product that may be evaluated in this article, or claim that may be made by its manufacturer, is not guaranteed or endorsed by the publisher.
